# Mesenchymal Stem Cell-Derived Exosomes Ameliorate Doxorubicin-Induced Cardiotoxicity

**DOI:** 10.3390/ph17010093

**Published:** 2024-01-10

**Authors:** Sawdah A. Ali, Dinender K. Singla

**Affiliations:** Division of Metabolic and Cardiovascular Sciences, Burnett School of Biomedical Sciences, College of Medicine, University of Central Florida, Orlando, FL 32816, USA; sawdah.ali@ucf.edu

**Keywords:** MSC-Exos, DOX, pyroptosis, inflammation

## Abstract

Doxorubicin (DOX) is an incessantly used chemotherapeutic drug that can cause detrimental dose-dependent effects such as cardiotoxicity and congestive heart failure. Hence, there is a need to discover innovative therapeutic approaches to counteract DOX-induced cardiotoxicity (DIC). MSC-Exos have shown to reduce apoptosis and cardiac fibrosis and promote cardiomyocyte proliferation in myocardial infracted mice. However, the effect of MSC-Exos on ameliorating DOX-induced pyroptosis has not been investigated. In this current study, H9c2 were first exposed to DOX to stimulate pyroptosis, followed by subsequent treatment with MSC-Exos, with further analysis performed through immunocytochemistry, western blotting, and RT-PCR. Our data depicted that post-treatment with MSC-Exos significantly (*p* < 0.05) reduced the HMGB1/TLR4 axis, inflammasome formation (NLRP3), pyroptotic markers (caspase-1, IL-1β, and IL-18), and the pyroptotic executioner (GSDMD) in DOX-treated H9c2 cells. In conclusion, our data show that MSC-Exos attenuates inflammation-induced pyroptosis in our in vitro DIC model. Our findings indicate that MSC-Exos may serve as a promising therapeutic intervention for mitigating DIC, as they maintain the therapeutic capabilities of MSCs while circumventing the drawbacks associated with traditional stem cell therapy.

## 1. Introduction

Doxorubicin (DOX) is an anthracycline antibiotic isolated from *Streptomyces peucetius caesius* that attained US Food and Drug Administration (FDA) approval in 1974 [1]. Following its approval, DOX became one of the most widely prescribed chemotherapeutic agents due to its broad-spectrum activity against both solid and metastatic cancers such as breast cancer, lymphomas, and leukemias [1,2]. Following entry into a cancer cell, DOX inhibits tumor progression through three purported mechanisms. Firstly, it binds to the 26S proteasome found in the cytoplasm enabling its entry into the nucleus, where it intercalates with the DNA resulting inhibition of DNA replication and transcription; secondly, once in the nucleus, it also intercalates with topoisomerase II disrupting DNA repair; and lastly, in the cytoplasm, DOX is oxidized to DOX-semiquinone which results in the production of free radicals such as reactive oxygen species (ROS) and disruption of the cell membrane [3,4].

Despite DOX’s broad-spectrum activity, its clinical use is limited due to both its detrimental dose-dependent and cumulative dose, dosage surpassing 400–700 mg/m^2^ for adults and 300 mg/m^2^ for children, effects such as DOX-induced cardiotoxicity (DIC), heart failure, and secondary malignancies [1,5]. DIC clinically presents as left ventricular dysfunction, arrhythmias, primarily atrial fibrillation, and congestive heart failure [6,7]. Consequently, the underlying mechanisms involved in DIC have been extensively researched. Multiple studies have suggested that the three major sources of cell damage in DIC are (1) mitochondrial dysfunction, (2) increased oxidative stress resulting in excessive production of reactive oxygen species (ROS), and (3) topoisomerase 2β inhibition resulting in multiple double-stranded breaks [1,6]. Additionally, studies have also shown the involvement of both regulated and unregulated cell death pathways, autophagy, necroptosis, ferroptosis, pyroptosis, and apoptosis, in DIC [6,8,9]. Therefore, it is believed that the molecular pathogenesis of DIC is multi-factorial with concurrent triggering of cell death pathways [1,6,10].

As previously mentioned, pyroptosis is one of the cell death pathways implicated in the pathogenesis of DIC [9]. Pyroptosis is a form of inflammation-mediated cell death that is characterized by the activation of the inflammasome and maturation of pro-inflammatory cytokines [11]. It is a distinct form of regulated cell death in that the pore executioner is gasdermin D (GSDMD), which leads to nuclear condensation, cell swelling, and membrane rupture [12]. Furthermore, pyroptosis is activated by pro-inflammatory cytokines, pathogen-associated molecular patterns (PAMPs), and damage-associated molecular patterns (DAMPs) [1]. DOX administration has been demonstrated to lead to an increased expression of high-mobility group box 1 (HMGB1), a known DAMP, that interacts with toll-like receptor 4 (TLR4), a receptor shown to play a key role in DOX-induced inflammation [13,14]. Following TLR4 activation, the nuclear factor kappa-light-chain-enhancer of activated B cells (NF-κB) is triggered, leading to the formation of the “NOD-like” receptor pyrin domain containing 3 (NLRP3) inflammasome, which activates caspase-1 [11,13]. Activated caspase-1 then cleaves pro-IL-1β, pro-IL-18, and gasdermin D (GSDMD), leading to the maturation of pro-inflammatory cytokines and formation of the GSDMD pore, a key executioner of pyroptosis [9,15].

As a result, there is a need for discovering novel therapeutic approaches to counteract the cardiotoxic effects of DOX. With this aim, numerous studies have investigated stem cells as potential cell-based therapies for DIC [16]. Embryonic stem cells (ESCs) have been evaluated due to their ability to differentiate into cardiomyocytes, which can be implanted in the heart to improve heart function. However, there are associated bioethical restrictions regarding the use of ESCs [16]. An alternative cell-based strategy researched is induced pluripotent stem cells (iPSCs), as they do not have ethical restrictions like ESCs but can be tumorigenic and are genetically unstable [17,18]. As a result, further studies have identified adult mesenchymal stem cells (MSCs) as a suitable tool for both regenerative and preventative therapy for DIC [16]. MSCs exert their cardioprotective effects through paracrine secretion of various growth factors, cytokines, and chemokines that aid in cardiac regeneration and repair [19]. Nonetheless, MSCs have their own disadvantages, such as weak myocardial homing, teratoma formation, and decreased viability following transplantation [19]. However, numerous studies have shown that the therapeutic potential of MSCs is mainly exerted by their paracrine effects, for example, by paracrine factors such as exosomes [20].

Exosomes are nanosized extracellular vesicles (30–200 nm diameter) that are generated through the endocytic pathway, released by exocytosis, dependent on their cell origin, and that carry a variety of constituents such as DNA, microRNA (miRNA), proteins, lipids, and metabolites [21]. MSCs-derived exosomes (MSC-Exos) preserve the therapeutic properties of MSCs without the associated disadvantages of stem cell therapy. MSC-Exos avoid the risk of becoming tumorigenic as they are not self-replicating, have biocompatibility and low immunogenicity, and an adequate number can be attained since they can be continuously secreted from immortalized cells [20,22]. Furthermore, MSC-Exos have shown to reduce apoptosis and cardiac fibrosis in myocardial-infracted mice, promote angiogenesis, and promote cardiomyocyte proliferation [23,24]. Moreover, a previous investigation revealed that pre-treatment with small extracellular vesicles derived from MSCs mitigated DOX-induced apoptosis by upregulating survivin [25]. However, whether MSC-Exos can attenuate DOX-induced proptosis in an in vitro model has never been reported. Consequently, an in-depth exploration of the influence of MSC-derived extracellular vesicles on the multiple regulated cell death pathways DIC may offer valuable insights into elucidating the potent therapeutic capabilities of MSC-Exos as a cell-free therapeutic modality.

Therefore, the current study aims to investigate the therapeutic potential of MSC-Exos in attenuating DOX-induced pyroptosis in cardiomyocytes. This leads to the identification of a novel therapeutic approach that can be effective at countering the cardiotoxicity induced by DOX.

## 2. Results

### 2.1. MSC-Exo Confirmation and Characterization

**Figure 1:**
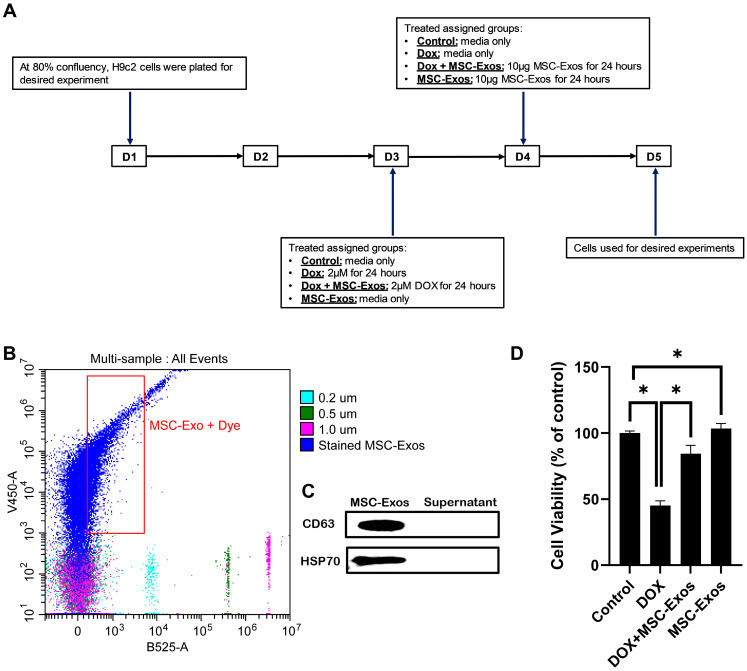
MSC-Exos characterization and ability to alleviate DOX-induced reduction in cell viability. (**A**) Schematic representation of the study design. (**B**) Flow cytometry analysis of violet-labeled isolated MSC-Exos (black box) and green-labeled sizing beads. (**C**) Western blot confirmation of exosome protein biomarkers CD63 and HSP70. (**D**) MSC-Exos reversed the reduction in cell viability induced by DOX, as assessed using MTT assay. The error bar denotes the standard mean error (SEM). * *p*  <  0.05.

To characterize the isolated MSC-Exos, flow cytometric size analysis was performed [26]. Isolated exosomes were stained with CellTrace™Violet and established to be less than 0.2 µm in size (Figure 1A), thereby confirming the presence of exosomes in the isolated pellet. Following flow cytometric analysis, further confirmation was executed through western blot analysis of exosome proteins CD63 and HSP70 as previously established [9]. Our representative data (Figure 1B) depict a prominent band for CD63 and HSP70 in the MSC-Exos pellet in comparison to the supernatant, therefore confirming the presence of exosomes in the isolated pellet.

### 2.2. Effect of MSC-Exos on Cell Viability of DOX-Treated H9c2 Cells

To determine the effect of MSC-Exos on DOX-treated H9c2 cells, cell viability was measured. In accordance with previous studies [27], DOX treatment significantly reduced the viability of H9c2 cells. However, post-treatment with MSC-Exos resulted in a significant (*p* < 0.05) increase in cell viability as compared to the DOX group (Figure 1D). Additionally, MSC-Exos control did not decrease cell viability; instead, it increased the cell viability in comparison to control. These results are indicative of MSC-Exo’s potential in restoring the DOX-suppressed cell viability.

### 2.3. MSC-Exos Diminishes Pyroptotic HMGB1 in an In Vitro DIC Model

**Figure 2:**
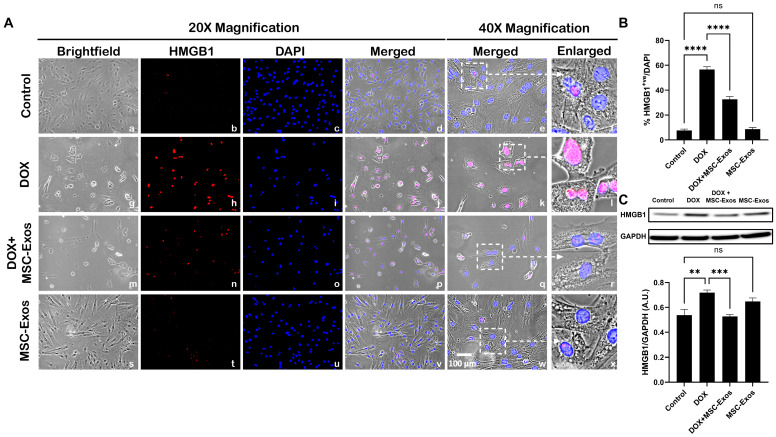
MSC-Exos diminishes pyroptotic initiator HMGB1 in an in vitro DIC model. (**A**) Representative photomicrographs depict 20× brightfield snapshots (a, g, m, s), HMGB1^+ve^ cells (red; b, h, n, t), DAPI (blue; c, i, o, u), and merged 20× snapshots (d, j, p, v). Brightfield merged snapshots at 40× (e, k, q, w) and white dotted boxes with arrows denote an expanded area of 40× snapshots (f, l, r, x). Scale bar = 100 µm. (**B**) MSC-Exos reduces the proportion of HMGB1^+ve^ cells induced by DOX. (**C**) HMGB1 was assessed in cell lysates, followed by densitometric analysis. The error bar denotes SEM. ** *p*  <  0.002, *** *p*  <  0.0006, **** *p*  <  0.0001, ns = non-significant.

To determine whether MSC-Exos attenuates DOX-induced upregulation of the pyroptotic initiator HMGB1 [14,28], we employed immunocytochemistry (ICC) staining. The representative photomicrographs (Figure 2A) depict a prominent increase in HMGB1 expression in the DOX-treated cells (g–l) when in comparison to the control group (a–f), and this increased expression was seen to notably reduce in the DOX + MSC-Exos group (m–r). In addition to this, MSC-Exos control (s–x) did not result in a significant increase in HMGB1. Our quantitative data demonstrate the significant (*p* < 0.0001) increase in HMGB1^+ve^ cells in the DOX group compared to the control, along with a significant (*p* < 0.0001) reduction in the DOX + MSC-Exos group (Figure 2B). To reinforce our immunofluorescence findings, western blot analysis was performed. A significant (*p* < 0.002) increase in HMGB1 protein expression was observed in the DOX group in comparison to the control group, while a significant (*p* < 0.0006) decrease was observed in the DOX + MSC-Exos group (Figure 2C). In addition, there was no significant difference between the control and MSC-Exos control groups. These results are indicative of MSC-Exo’s potential in diminishing DOX-induced upregulation of HMGB1.

### 2.4. MSC-Exos Reduces TLR4 Expression in an In Vitro DIC Model

**Figure 3:**
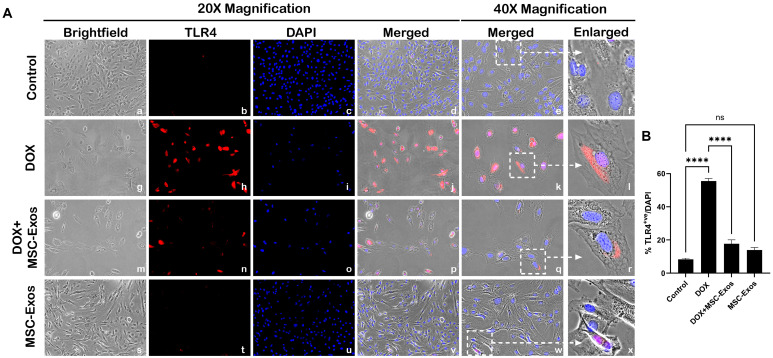
MSC-Exos reduced TLR4 expression an in vitro DIC model. (**A**) Representative photomicrographs depict 20× brightfield snapshots (a, g, m, s), TLR4^+ve^ cells (red; b, h, n, t), DAPI (blue; c, i, o, u), and merged 20× snapshots (d, j, p, v). 40× brightfield merged snapshots (e, k, q, w) and white dotted boxes with arrows denote an expanded area of 40× snapshots (f, l, r, x). Scale bar = 100 µm. (**B**) MSC-Exos reduces the pyroptotic proportion of TLR4^+ve^ cells induced by DOX. The error bar denotes SEM. **** *p*  <  0.0001, ns = non-significant.

Previously published studies have shown that HMGB1, a DAMP, can activate various intracellular signaling pathways by interacting with TLR4 [14,28,29] hence we investigated nuclear/perinuclear TLR4 expression via ICC staining as previously published [9,30,31]. From the representative photomicrographs (Figure 3A), a noticeable increase in TLR4^+ve^ cells is observed in the DOX group (g–l) as compared to the control group (a–f). Further, MSC-Exos post-treatment resulted in a pronounced reduction in TLR4^+ve^ cells (m–r), and MSC-Exos control alone (s–x) did not result in a significant increase in TLR4 expression. Furthermore, the nuclear/perinuclear localization of TLR4 observed in our study corroborates with previously published studies on TLR4 nuclear/perinuclear expression [9,30,31]. Additional quantitative analysis (Figure 3B) showed a significant (*p* < 0.0001) increase in TLR4^+ve^ cells in the DOX group in comparison to the control along with a significant (*p* < 0.0001) reduction in the DOX + MSC-Exos group. Furthermore, no significant difference was observed between the control and MSC-Exos control groups. These results imply that MSC-Exos treatment can reduce DOX-induced upregulation of TLR4.

### 2.5. MSC-Exos Decreases NLRP3 Inflammasome Formation in an In Vitro DIC Model

**Figure 4:**
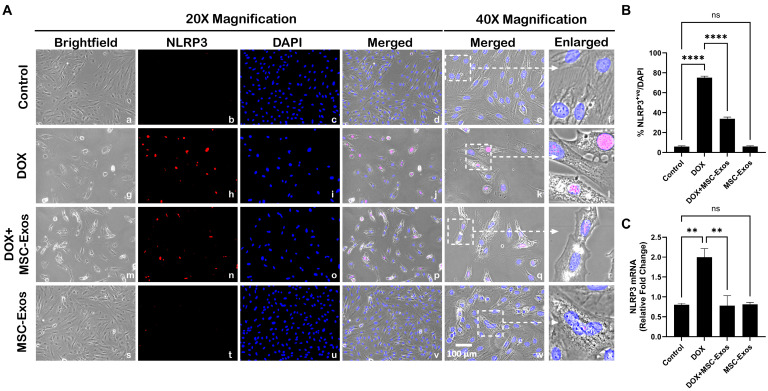
MSC-Exos decreases NLRP3 inflammasome formation in an in vitro DIC model. (**A**) Representative photomicrographs depict 20× brightfield snapshots (a, g, m, s), NLRP3^+ve^ cells (red; b, h, n, t), DAPI (blue; c, i, o, u), and merged 20× snapshots (d, j, p, v). 40× brightfield merged snapshots (e, k, q, w) and white dotted boxes with arrows denote an expanded area of 40× snapshots (f, l, r, x). Scale bar = 100 µm. (**B**) MSC-Exos reduces the proportion of NLRP3^+ve^ cells induced by DOX. (**C**) Relative fold change of NLRP3. The error bar denotes SEM. ** *p*  <  0.002, **** *p*  <  0.0001, ns = non-significant.

Our lab has formerly disseminated the involvement of the NLRP3 inflammasome as a downstream effector of pyroptosis [9,29]. Thus, we performed ICC staining to evaluate NLRP3 inflammasome formation. The photomicrographs (Figure 4A) show a high number of NLRP3^+ve^ cells in the DOX group (g–l) as compared to the control group (a–f). This increase in NLRP3^+ve^ cells was prominently reduced following MSC-Exos treatment (m–r), and MSC-Exos control alone (s–x) did not result in a significant increase in NLRP3^+ve^ cells. Our quantitative analysis (Figure 4B) showed a significant (*p* < 0.0001) increase in NLRP3^+ve^ cells in the DOX group compared to the control along with a significant (*p* < 0.0001) reduction in the DOX + MSC-Exos group. To corroborate our immunofluorescence findings, RT-PCR was performed to evaluate NLRP3 gene expression. A significant (*p* < 0.002) increase in NLRP3 mRNA was detected in the DOX group in comparison to the control group, while a significant (*p* < 0.002) decrease was observed in the DOX + MSC-Exos group (Figure 4C). Moreover, there was no significant difference between the control and MSC-Exos control groups. These findings suggest that the administration of MSC-Exos has the potential to decrease DOX-induced upregulation of the NLRP3 inflammasome.

### 2.6. MSC-Exos Mitigates Pyroptotic Cascade Markers Caspase-1, IL-1β, and IL-18 in an In Vitro DIC Model

**Figure 5:**
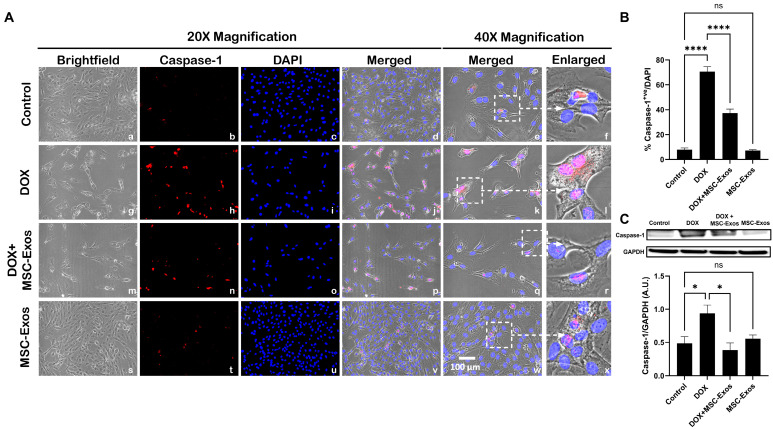
MSC-Exos mitigates pyroptotic cascade marker caspase-1 in an in vitro DIC model. (**A**) Representative photomicrographs depict 20× brightfield snapshots (a, g, m, s), caspase-1^+ve^ cells (red; b, h, n, t), DAPI (blue; c, i, o, u), and merged 20× snapshots (d, j, p, v). 40× brightfield merged snapshots (e, k, q, w) and white dotted boxes with arrows denote an expanded area of 40× snapshots (f, l, r, x). Scale bar = 100 µm. (**B**) MSC-Exos reduces the proportion of caspase-1^+ve^ cells induced by DOX. (**C**) Caspase-1 was assessed in cell lysates, followed by densitometric analysis. The error bar denotes SEM. * *p*  <  0.05, **** *p*  <  0.0001, ns = non-significant.

Studies have shown that following NLRP3 formation, caspase-1 is activated, resulting in the activation of downstream pro-inflammatory cytokines IL-1β and IL-18 [9,29]. Therefore, to first understand whether MSC-Exos treatment mitigates caspase-1, ICC staining was performed. The representative photomicrographs show a high number of caspase-1^+ve^ cells along with a significant increase (*p* < 0.0001) in of caspase-1^+ve^ cells (Figure 5B) in the DOX group (g–l) in comparison to the control group (a–f). Further to this, MSC-Exos post-treatment resulted in a decrease in the expression of caspase-1^+ve^ cells (Figure 5A; m–r) along with a significant (*p* < 0.0001) reduction in caspase-1^+ve^ cells (Figure 5B). To verify our immunofluorescence findings, a western blot was performed to evaluate caspase-1 and IL-1β protein expression. A significant (*p* < 0.05) increase in caspase-1 (Figure 5C) protein expression was detected in the DOX group in comparison to the control group, while a significant (*p* < 0.05) decrease was observed in the DOX + MSC-Exos group. In addition, no significant difference in caspase-1 was observed between the control and MSC-Exos control groups.

**Figure 6:**
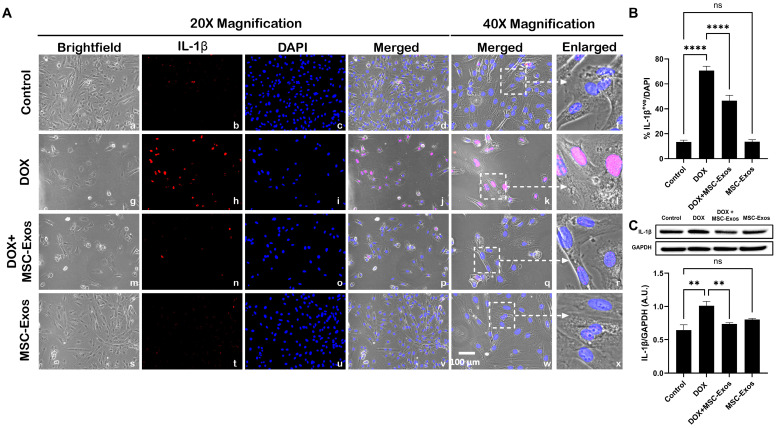
MSC-Exos mitigates pyroptotic cascade marker IL-1β in an in vitro DIC model. (**A**) Representative photomicrographs depict 20× brightfield snapshots (a, g, m, s), IL-1β^+ve^ cells (red; b, h, n, t), DAPI (blue; c, i, o, u), and merged 20× snapshots (d, j, p, v). 40× brightfield merged snapshots (e, k, q, w) and white dotted boxes with arrows denote an expanded area of 40× snapshots (f, l, r, x). Scale bar = 100 µm. (**B**) MSC-Exos reduces the proportion of IL-1β^+ve^ cells induced by DOX. (**C**) IL-1β was assessed in cell lysates, followed by densitometric analysis. The error bar denotes SEM. ** *p*  <  0.002, **** *p*  <  0.0001, ns = non-significant.

Following the evaluation of caspase-1, downstream marker IL-1β was investigated through ICC staining. The representative photomicrographs show a high quantity of IL-1β^+ve^ cells (Figure 6A) along with a significant increase (*p* < 0.0001) in IL-1β^+ve^ cells (Figure 6B) in the DOX group (g–l) in comparison to the control group (a–f). Moreover, MSC-Exos post-treatment resulted in a decrease in the expression of IL-1β^+ve^ cells (Figure 6A; m–r) along with a significant (*p* < 0.0001) reduction in IL-1β^+ve^ cells (Figure 6B). To confirm our immunofluorescence findings, a western blot was performed to evaluate IL-1β protein expression. A significant (*p* < 0.002) increase in IL-1β (Figure 6C) protein expression was detected in the DOX group in comparison to the control group, while a significant decrease (*p* < 0.002) was observed in the DOX + MSC-Exos group. In addition, no significant difference in IL-1β was observed between the control and MSC-Exos control groups.

**Figure 7:**
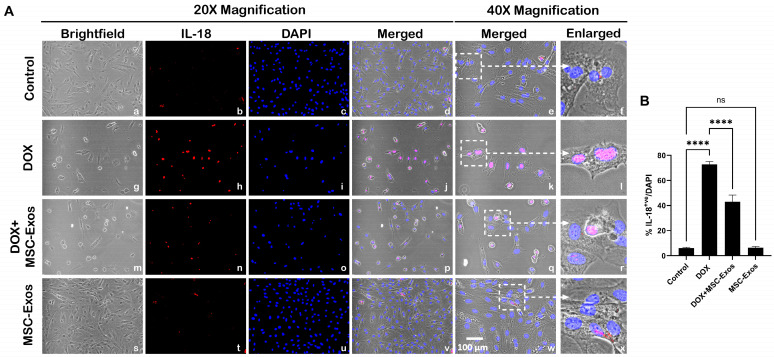
MSC-Exos mitigates pyroptotic cascade marker IL-18 in an in vitro DIC model. (**A**) Representative photomicrographs depict 20× brightfield snapshots (a, g, m, s), IL-18^+ve^ cells (red; b, h, n, t), DAPI (blue; c, i, o, u), and merged 20× snapshots (d, j, p, v). 40× brightfield merged snapshots (e, k, q, w) and white dotted boxes with arrows denote an expanded area of 40× snapshots (f, l, r, x). Scale bar = 100 µm. (**B**) MSC-Exos reduces the proportion of IL-18^+ve^ cells induced by DOX. The error bar denotes SEM. **** *p*  <  0.0001, ns = non-significant.

Subsequently, downstream marker IL-18 was investigated through ICC staining. The representative photomicrographs show a high number of IL-18^+ve^ cells (Figure 7A) along with a significant increase (*p* < 0.0001) in IL-18^+ve^ cells (Figure 7B) in the DOX group (g–l) as compared to the control group (a–f). Further to this, MSC-Exos post-treatment resulted in a decrease in the expression of IL-18^+ve^ cells (Figure 7A; m–r) along with a significant (*p* < 0.0001) reduction in IL-18^+ve^ cells (Figure 7B). Also, no significant difference in IL-18 was observed between the control and MSC-Exos control groups. Overall, these findings indicate that MSC-Exos administration has the potential to mitigate DOX-induced upregulation of the pyroptotic cascade.

### 2.7. MSC-Exos Attenuates Pyroptotic Executioner GSDMD in an In Vitro DIC Model

**Figure 8:**
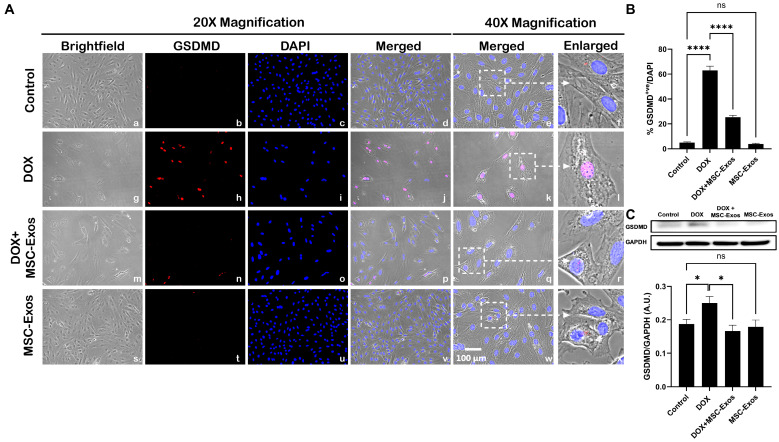
MSC-Exos attenuates pyroptotic executioner GSDMD in an in vitro DIC model. (**A**) Representative photomicrographs depict 20× brightfield snapshots (a, g, m, s), GSDMD^+ve^ cells (red; b, h, n, t), DAPI (blue; c, i, o, u), and merged 20× snapshots (d, j, p, v). 40× brightfield merged snapshots (e, k, q, w) and white dotted boxes with arrows denote an expanded area of 40× snapshots (f, l, r, x). Scale bar = 100 µm. (**B**) MSC-Exos reduces the proportion of GSDMD^+ve^ cells induced by DOX. (**C**) GSDMD was assessed in cell lysates, followed by densitometric analysis. The error bar denotes SEM. * *p*  <  0.05, **** *p*  <  0.0001, ns = non-significant.

Studies have shown that pyroptotic executioner GSDMD is required for pyroptosis [15,32]. Thus, we performed ICC staining to investigate GSDMD expression. Our photomicrographs (Figure 8A) depict a prominent increase in GSDMD^+ve^ cells in the DOX-treated cells (g–l) when compared to the control group (a–f), and this increased expression was seen to notably reduce in the DOX + MSC-Exos group (m–r). In addition to this, MSC-Exos alone (s–x) did not result in a substantial increase in GSDMD^+ve^ cells. Our quantitative data demonstrate the significant (*p* < 0.0001) increase in GSDMD^+ve^ cells in the DOX group compared to the control along with a significant (*p* < 0.0001) reduction in the DOX + MSC-Exos group (Figure 8B). To strengthen our immunofluorescence findings, western blot analysis was performed. A significant (*p* < 0.05) increase in GSDMD protein expression was observed in the DOX group in comparison to the control group, while a significant (*p* < 0.05) decrease was observed in the DOX + MSC-Exos group (Figure 8C). Further to this, there was no significant difference between the control and MSC-Exos control groups. This set of data suggests that treatment with MSC-Exos can potentially attenuate DOX-induced upregulation of the pyroptotic executioner GSDMD.

## 3. Discussion

Doxorubicin-induced cardiotoxicity (DIC) is multifaceted in nature, encompassing not only mitochondrial dysfunction and production of ROS but also involvement of multiple cell death pathways. In the early stages of DOX therapy, asymptomatic and symptomatic cardiomyopathy highlighted by epigenetic changes and cellular injury presents and then later develops into abnormal cell signaling, resulting in cardiac dysfunction, fibrosis, endothelial injury, and hypotensive remodeling [33]. Altogether, these events result in congestive heart failure, a detriment that is of mounting concern as cancer survivors are increasing in number.

In recent years, novel exosome therapeutics aimed at cardiac regeneration and repair have garnered interest. Traditionally, strategies employed to reduce DIC is to alter the delivery mechanism of DOX by encapsulating it in a liposome, therefore changing its biodistribution. However, this limits the use to specific cancers due to its high cost [7]. Alternatively, a drug that targets topoisomerase IIβ enzyme and reduces iron-mediated production of oxidative radicals, like the FDA-approved drug dexrazoxane, has been used to counter DIC. However, it has associated restrictions because it was seen to increase the incidence of secondary malignancies [7]. Furthermore, various cell-based therapies, such as stem cells, have been investigated. Nonetheless, there are constraints in their use due to the associated teratoma formation, weak myocardial homing, and weak viability following transplantation [23]. Hence, employing a cell-free alternative that retains the advantages associated with stem cells, such as stem cell-derived exosomes, would present a novel therapeutic intervention that would have greater efficacy in countering DIC.

Mesenchymal stem cell-derived exosomes (MSC-Exos) secrete diverse factors that result in anti-inflammatory, pro-angiogenic, anti-apoptotic, and anti-fibrotic outcomes following intravenous and intramyocardial transplantation [34]. Furthermore, a prior study has identified that pre-treatment with MSC-derived small extracellular vesicles attenuated DOX-induced apoptosis through upregulation of survivin [25]. Therefore, further investigation into the impact of MSC-Exos on the multifaceted regulated cell death pathways implicated in DIC could contribute to uncovering the robust therapeutic potential of MSC-Exos as a cell-free therapeutic.

In this study, we explored the therapeutic potential of MSC-Exos, a cell-free alternative to MSCs, in attenuation of pyroptosis (inflammation-mediated cell death) in our established in vitro DIC model. In this context, our investigation is the first to accentuate the ability of MSC-Exos to attenuate the HMBG1/TLR4 axis and NLRP3 inflammatory cascade.

DOX treatment mediates the upregulation of HMGB1, a DAMP and upstream activator of pyroptotic cell death. Further to this, HMGB1 is an extracellular molecule that triggers inflammatory responses under conditions such as sepsis and myocardial infarction and carries out its biological role through its receptor TLR4 [14,28]. Studies have depicted that DOX treatment specifically results in an increased secretion of HMGB1 in the heart and brain [14,35]. However, it is unknown whether MSC-Exos treatment would attenuate the DOX-induced upregulation of HMGB1. Data presented in the current study depicts that DOX treatment resulted in a heightened expression of HMGB1 in cardiomyocytes, thereby corroborating with other published studies on HMGB1 upregulation following DOX treatment [14,28,35]. Further to this, MSC-Exos treatment resulted in a substantial reduction in HMGB1 in comparison to the DOX group. A justification behind MSC-Exos’s ability to attenuate the expression of HMGB1 lies in substantiated studies indicating the anti-inflammatory effects of MSC-Exos along with its cardioprotective potential [34].

As aforementioned, the receptor for HMGB1 is TLR4, a constituent of the innate immune system that belongs to the TLR family and thereby responds to both endogenous and exogenous signals, thereby triggering various pathophysiological functions such as pyroptotic cell death upstream of NLRP3 [13]. Furthermore, TLR4 is not only expressed in immune cells but is also expressed in the heart and brain following DOX administration [9,13,36]. Henceforth, to further understand the significance of HMGB1 downregulation upon MSC-Exos administration, we assessed TLR4. Our data validate that elevated HMGB1 results in an upregulation of TLR4, while MSC-Exos treatment substantially reduced levels, indicating the ability of MSC-Exos to inhibit TLR4. MSC-Exos is known to release microRNAs that can inhibit TLR4 signaling [37]. Hence, the reduced TLR4 expression observed following MSC-Exos treatments could be a result of decreased HMGB1 expression, thereby reducing TLR4 activation as previously mentioned or directly due to factors released from the MSC-Exos.

TLR4 signaling activates the NLRP3 inflammasome, a mediator of pyroptotic cell death. NLRP3, pro-caspase-1, and apoptosis-associated speck-like protein containing a CARD (ASC) constitutes the NLRP3 inflammasome which self-cleaves to form active caspase-1 [9,29]. Therefore, further, we investigated the effect of MSC-Exos on NLPR3 and caspase-1 after DOX treatment, aimed to strengthen the validation of MSC-Exos’ therapeutic efficacy in regulating the HMGB1/TLR4/NLRP3 inflammatory cascade. Our data exhibit that DOX treatment increases the expression of NLRP3 and caspase-1, thereby agreeing with previously published data, while MSC-Exos reduced these levels.

Additionally, caspase-1 cleaves and initiates pro-inflammatory cytokines pro-IL-1β and pro-IL-18 to their active form, IL-1β and IL-18, and GSDMD to N-GSDMD, the pyroptotic executioner that forms membrane pores through which the pro-inflammatory cytokines are released [9,36]. Our data demonstrate that MSC-Exos reduced the DOX-induced upregulation of IL-1β, IL-18, and GSDMD. Taken together, these findings indicate for the first time that MSC-Exos targets DOX-induced HMGB1 upregulation, thereby attenuating the downstream pyroptotic cascade.

In conclusion, our study demonstrates for the first time that MSC-Exos attenuates DOX-induced (1) HMGB1/TLR4 axis, (2) NLRP3 inflammatory cascade, and (3) GSDMD pore formation, resulting in diminished pyroptosis. These findings indicate a decrease in HMGB1 and a further reduction in pyroptosis, suggesting that MSC-Exos attenuates the upstream HMGB1/TLR4 axis. However, the possibility that MSC-Exos could have a potential direct effect on the pyroptotic pathways (caspase-1, IL-1β, IL-18) and pyroptotic executioner GSDMD needs to be further explored. Further to this, this study utilizes an established in vitro model designed to mimic in vivo conditions. However, validating these findings through examinations with isolated cardiomyocytes and human induced pluripotent stem cell (iPSC)-derived cardiomyocytes would enrich the depth of our understanding. Additionally, substantiating these findings in a mouse in vivo model is imperative and holds relevance for pre-clinical investigations. A comprehensive elucidation of the effects of MSC-Exos within these distinct models significantly broadens the scope and relevance of our research. Additionally, our study corroborates with established studies that illustrate the therapeutic potential of MSC-Exos in DIC, myocardial infarction, and heart failure models [23,25,34,37]. However, prospective investigations are required to identify the specific factors within MSC-Exos that contribute to its therapeutic effects. Moreover, this study provides preliminary evidence that MSC-Exos can be a prospective therapeutic agent to counter DOX-induced cardiotoxicity as it preserves the potential of MSCs without the associated disadvantages of stem cell therapy.

## 4. Materials and Methods

### 4.1. Isolation of Mesenchymal Stem Cell-Derived Exosomes

C57BL/6 mouse bone MSCs were purchased from OriCell (Roseland, NJ, USA). Briefly, MSCs were cultured with mouse bone marrow mesenchymal stem cells complete medium (OriCell, Roseland, NJ, USA). MSCs were cultured in a T75 flask with complete medium for 48 h, then replaced with serum-free knockout Dulbecco’s Modified Eagle Medium (DMEM; ThermoFisher Scientific, Waltham, MA, USA) and cells were grown for an additional 48 h. Next, the culture medium was collected and centrifuged, and the supernatant was collected in sterile tubes and combined with Exoquick-TC Exo precipitation solution [System Biosciences (SBI), Palo Alto, CA, USA] in accordance with the manufacturer’s instruction. Following incubation, tubes were centrifuged, supernatant was aspirated, and exosome pellet was collected.

### 4.2. MSC Exosome Confirmation and Characterization

Isolated Exos were confirmed via flow cytometry analysis with CellTrace™ Violet (ThermoFisher Scientific, Waltham, MA, USA) as previously published [26]. In brief, isolated Exos were incubated with CellTrace reagent for 20 min at 37 °C. Following labeling, flow cytometry analysis was accomplished using the CytExpert Software v2.5 for the CytoFLEX Flow Cytometer (Beckman Coulter, Brea, CA, USA).

Exo pellet was then characterized through a western blot for specific protein biomarkers for exosomes, namely CD63 and heat shock protein 70 (HSP70), as published [9]. In brief, exosomal proteins were extracted by reconstitution with radioimmunoprecipitation assay lysis buffer (RIPA), and the concentration of isolated protein was acquired using the Bio-Rad protein assay. The extracted protein (50 μg) and ladder (SeeBlue Plus2 Prestained Standard) [38] were loaded onto 4–12% Bolt gels and ran for 22 min at 200 V, followed by transfer to a polyvinylidene difluoride (PVDF) membrane using the iBlot2 Gel Transfer Device. Membranes were then incubated with antibodies CD63 (1:1000; SBI, Palo Alto, CA, USA) and HSP70 (1:1000; SBI, Palo Alto, CA, USA).

### 4.3. H9c2 Cell Culture

Rat embryonic cardiomyocyte cells (H9c2) purchased from American Type Culture Collection (ATCC) were maintained in cell culture flasks with DMEM (ThermoFisher Scientific, Waltham, MA, USA) supplemented with 10% fetal bovine serum (FBS; R&D Systems, Minneapolis, MN, USA), penicillin/streptomycin (P/S; ThermoFisher Scientific, Waltham, MA, USA), sodium pyruvate (NaP; ThermoFisher Scientific, Waltham, MA, USA), glutamine (ThermoFisher Scientific, Waltham, MA, USA), and non-essential amino acids (NEAA, ThermoFisher Scientific, Waltham, MA, USA) at 37 °C in the presence of 5% CO_2_.

### 4.4. MTT Assay

H9c2 cells were cultured in 96-well plates (10,000 cells/well) until ≈60–80% and then divided into 4 groups: (1) control, (2) DOX, (3) DOX + MSC-Exos, and (4) MSC-Exos (exosome control). Briefly, group 1 and group 4 H9c2 cells were cultured in growth media, while group 2 and 3 cells were treated with 2μM DOX (prepared in sterilized water) for 24 h. Following the 24-hour incubation, media from all groups were removed. Subsequently, groups 1 and 2 cells received fresh growth media, while groups 3 and 4 cells received 10 μg of MSC-Exos, and cells were cultured for an additional 24 h (Figure 1A). After the treatment period, MTT (Roche, Indianapolis, IN, USA) was performed following the manufacturer’s protocol as previously published [39]. Briefly, MTT reagent was added to each well for 4 h at 37 °C, resulting in the formation of formazan crystal, which was then solubilized and incubated overnight. Following the incubation, absorbance was measured at 550 nm and 650 nm (background) using the SpectraMAX i3 Multi-Mode Microplate Reader. Cell viability was calculated as a percent of control.

### 4.5. Immunocytochemistry (ICC) Staining

H9c2 cells were cultured in eight-chamber slides (10,000 cells/chamber) and treated as previously described (Figure 1A). Following treatment, slides were washed with sterile 1X phosphate-buffered saline (PBS), fixed with 4% paraformaldehyde, and permeabilized with 0.3% Triton X in the dark. Following permeabilization, slides were then blocked and incubated with primary antibodies: HMGB1 (1:250; Abcam, Waltham, MA, USA), TLR4 (1:250; Abcam Waltham, MA, USA), NLRP3 (1:250; LifeSpan BioSciences, Shirley, MA, USA), caspase-1 (1:250; Abcam, Waltham, MA, USA), IL-1β (1:250; Abcam, Waltham, MA, USA), IL-18 (1:250; Abcam, Waltham, MA, USA), and GSDMD (1:250; Abcam, Waltham, MA, USA) overnight at 4 °C. Following primary antibody incubation, cells were washed with 1X PBS and incubated with secondary antibody Alexa 568 (anti-rabbit, 1:1000; Invitrogen, Carlsbad, CA, USA) at room temperature (RT). Lastly, slides were washed and counterstained using Vectashield Antifade mounting medium containing 4′-6-diamidino-2-phenylindole (DAPI) to stain the nuclei and mounted with coverslips.

Images for quantification were taken using the 20× magnification, and representative images were taken using the 40× magnification on the BZ-X810 Keyence microscope. The percentage of pyroptotic cell death was determined by evaluating the number of positively stained cells (in red) over the total DAPI^+ve^ (in blue) multiplied by 100 [(total cells^+ve^/total DAPI^+ve^) × 100)] using the ImageJ software. Bar graphs were generated using GraphPad Prism software v10.

### 4.6. Western Blot

H9c2 cells were cultured in 100 mm^2^ dishes (500,000 cells/dish), adhering to the previously described treatment plans (Figure 1A). After the treatment period, cells were lysed using RIPA, and the resultant cell lysate was obtained. Bio-Rad protein assay was utilized to elucidate the concentration of isolated proteins. The protein samples (25 μg) and ladder (SeeBlue Plus2 Prestained Standard) were loaded onto 4–12% Bolt gels and ran for 22 min at 200 V, followed by transfer to a polyvinylidene difluoride (PVDF) membrane using the iBlot2 Gel Transfer Device as previously published [29]. Membranes were blocked with 5% non-fat milk and incubated overnight at 4 °C with primary antibodies (1:1000) specific for pyroptotic markers (HMGB1, caspase-1, IL-1β, and GSDMD), followed by incubation with secondary antibody (anti-rabbit horseradish peroxidase-conjugated antibody, 1:1000; Cell Signaling, Danvers, MA, USA) before imaging with the Sapphire Biomolecular Imager. Densitometric analyses were performed using Image J software v1.39o, and all bands were normalized to the loading control glyceraldehyde-3-phosphate dehydrogenase (GAPDH; Cell Signaling, Danvers, MA, USA) and expressed in arbitrary units (A.U.). The bar graphs were generated using GraphPad Prism software v10.

### 4.7. RT-PCR

H9c2 cells were cultured in 60 mm^2^ dishes (200,000 cells/dish), adhering to the previously described treatment plans (Figure 1A). After the treatment period, TRIzol reagent was utilized to extract total RNA, cDNA (SuperScript™ III First-Strand Synthesis SuperMix for qRT-PCR; ThermoFisher, Waltham, MA, USA) was reverse transcribed, and RT-PCR was performed to evaluate the gene expression levels for NLRP3 (Table 1) as previously described [29]. GAPDH was used as the housekeeping gene to normalize the fold expression. The bar graph was generated using GraphPad Prism software v10.

### 4.8. Statistical Analysis

Statistical significance was assessed at *p* < 0.05 through the analysis of data using both Student’s *t*-test and one-way ANOVA, followed by Tukey’s test for post hoc comparisons. Data representation in graphs was made using GraphPad Prism software v10, and values are presented as means ± SEM.

## Figures and Tables

**Table 1:** RT-PCR rat primers.

Table	Forward Primer	Reverse Primer
NLRP3	5′-GGTGACCTTGTGTGTGCTTG-3′	5′-ATGTCCTGAGCCATGGAAGC-3′
GAPDH	5′-GCCCACTAAAGGGCATCCTG-3′	5′-GAGTTGGGATGGGGACTCTCA-3′

## Data Availability

Data is contained within the article.
